# Menstrual cycle influence on cognitive function and emotion processing—from a reproductive perspective

**DOI:** 10.3389/fnins.2014.00380

**Published:** 2014-11-24

**Authors:** Inger Sundström Poromaa, Malin Gingnell

**Affiliations:** ^1^Department of Women's and Children's Health, Uppsala UniversityUppsala, Sweden; ^2^Department of Psychology, Uppsala UniversityUppsala, Sweden

**Keywords:** menstrual cycle, estradiol, progesterone, cognition, emotion, functional magnetic resonance imaging

## Abstract

The menstrual cycle has attracted research interest ever since the 1930s. For many researchers the menstrual cycle is an excellent model of ovarian steroid influence on emotion, behavior, and cognition. Over the past years methodological improvements in menstrual cycle studies have been noted, and this review summarizes the findings of methodologically sound menstrual cycle studies in healthy women. Whereas the predominant hypotheses of the cognitive field state that sexually dimorphic cognitive skills that favor men are improved during menstrual cycle phases with low estrogen and that cognitive skills that favor women are improved during cycle phases with increased estrogen and/or progesterone, this review has not found sufficient evidence to support any of these hypotheses. Mental rotation has gained specific interest in this aspect, but a meta-analysis yielded a standardized mean difference in error rate of 1.61 (95% CI −0.35 to 3.57), suggesting, at present, no favor of an early follicular phase improvement in mental rotation performance. Besides the sexually dimorphic cognitive skills, studies exploring menstrual cycle effects on tasks that probe prefrontal cortex function, for instance verbal or spatial working memory, have also been reviewed. While studies thus far are few, results at hand suggest improved performance at times of high estradiol levels. Menstrual cycle studies on emotional processing, on the other hand, tap into the emotional disorders of the luteal phase, and may be of relevance for women with premenstrual disorders. Although evidence at present is limited, it is suggested that emotion recognition, consolidation of emotional memories, and fear extinction is modulated by the menstrual cycle in women. With the use of functional magnetic resonance imaging, several studies report changes in brain reactivity across the menstrual cycle, most notably increased amygdala reactivity in the luteal phase. Thus, to the extent that behavioral changes have been demonstrated over the course of the menstrual cycle, the best evidence suggests that differences in sexually dimorphic tasks are small and difficult to replicate. However, emotion-related changes are more consistently found, and are better associated with progesterone than with estradiol such that high progesterone levels are associated with increased amygdala reactivity and increased emotional memory.

## Introduction

The menstrual cycle has attracted research interest ever since the 1930s (Frank, 1931). Despite the extensive research on this excellent and ecological model of ovarian steroid influence on emotion, behavior, and cognition, relatively few findings have emerged as conclusive. In fact, already in 1973 did Barbara Sommer review the existing literature (at that point 33 scientific papers were available) and concluded that no menstrual cycle-related changes in cognitive and perceptual-motor performance were evident (Sommer, 1973). Yet, she also concluded that methodological problems were common, specifically concerning menstrual cycle definition and hormonal state. With increasingly accessible methods for steroid hormone analyses, both in serum and saliva, tremendous improvements in menstrual cycle studies have been achieved over the past years. One example of this is premenstrual dysphoric disorder (PMDD), which used to be a loosely defined syndrome with numerous, but inadequate, treatment options ranging from herbal remedies, to vitamins and progestagens. With strict definitions in the Diagnostic and Statistical Manual of Mental Disorders, thorough menstrual cycle phase staging and high-quality randomized clinical trials, clinicians today are able to offer afflicted women effective and evidence-based treatments (Marjoribanks et al., 2013).

**Figure 1:**
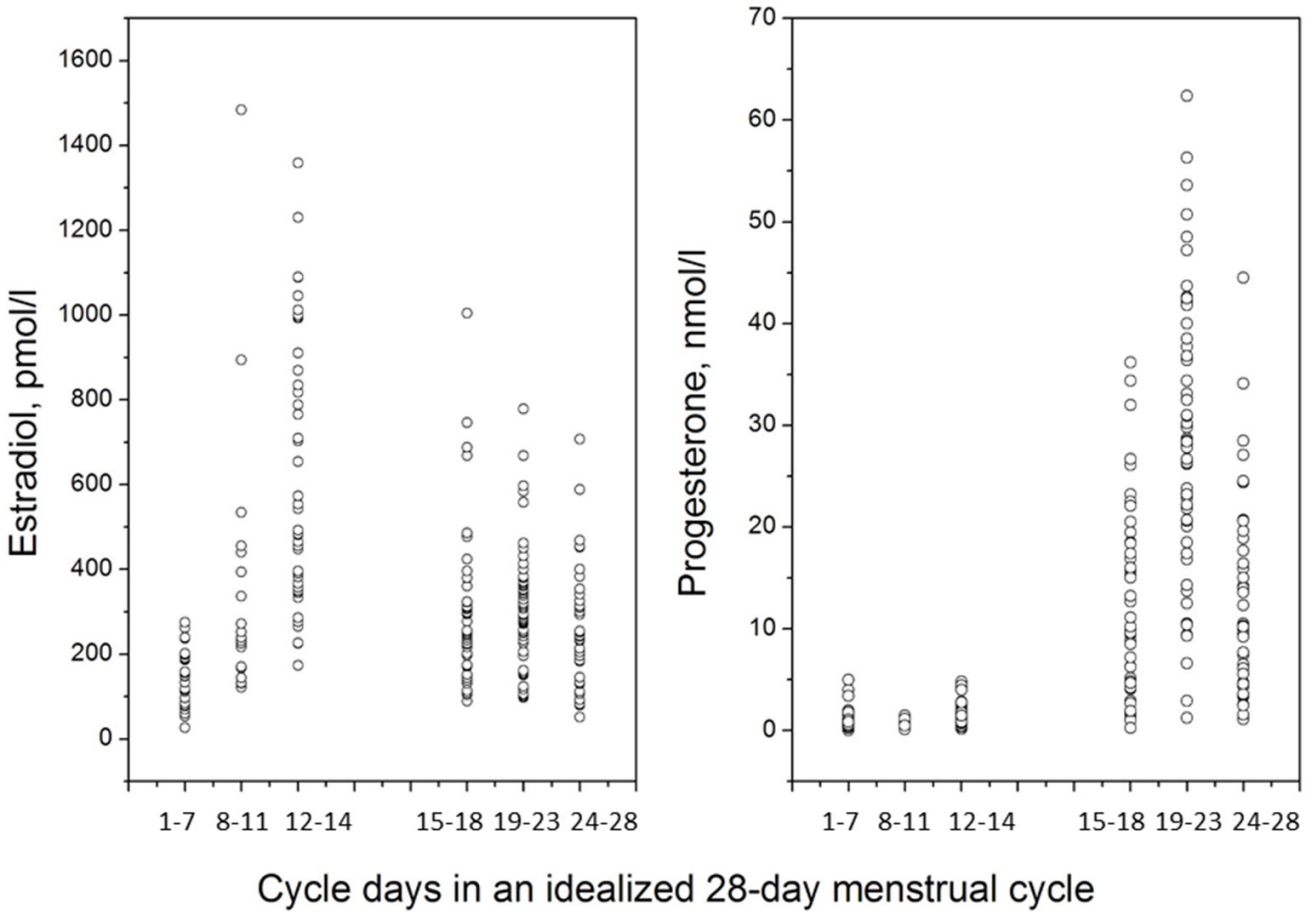
**Estradiol and progesterone levels across the menstrual cycle, frequently sampled in 47 healthy women, 18–42 years old, with self-reported history of regular menstrual cycles, and no hormonal use**. Cycle phase staging was accomplished by forward counting from onset of menstrual cycle (days 1–7), backward counting from day of LH surge (days 8–11, and days 12–14), forward counting from LH surge (days 15–18, and days 19–23) and backward counting from onset of next menses (days 24–28).

The idealized menstrual cycle consists of 28 days, but it is normal that cycle length varies between 21 and 35 days (Lenton et al., 1984a). The menstrual cycle length decreases with advancing age (Lenton et al., 1984a), and approximately 7% of menstrual cycles are shorter than 26 days (Brodin et al., 2008). Oligomenorrhea is defined as menstrual cycle length of 35 days or more (Treloar et al., 1967; Chiazze et al., 1968), and is in turn one of the criteria for the polycystic ovary syndrome (PCOS) (Rotterdam ESHRE/ASRM-Sponsored PCOS Consensus Workshop Group, 2004). In an infertility setting, which may not be entirely representative of the general population, 5.4% of menstrual cycles are 35 days or longer (Brodin et al., 2008). The follicular phase is characterized by follicular development, in response to increased levels of follicle stimulating hormone (FSH) in the early follicular phase, but later, as a dominant follicle has been selected the stimulatory need for FSH gradually diminishes. The pool of growing follicles progressively increase their estradiol production, but the pre-ovulatory estradiol surge is, in fact, a direct signal from the dominant follicle to the hypothalamus that it is ready for the final events leading up to ovulation (Speroff and Fritz, 2010). Typically, the first seven days of the menstrual cycle (in this review denoted as the early follicular phase) are characterized by low serum levels of estradiol, around or below 200 pmol/l, but during the first days of menses it is not uncommon to encounter estradiol serum concentrations in the postmenopausal range, i.e., below 100 pmol/l (Figure 1). With the rise of a dominant follicle, estradiol levels rapidly increase during the second week of the menstrual cycle (late follicular phase), and during the pre-ovulatory estradiol surge levels between 600 and 2500 pmol/l, or even higher, may be encountered (Schuster et al., 2010). The estradiol peak is followed 12–24 h later by the luteinizing hormone (LH) surge, and ovulation, in turn, occurs typically 10–12 h after the LH surge (Speroff and Fritz, 2010). The LH surge can be measured by urinary LH kits, and it is generally advised to start daily tests already on menstrual cycle day 10, in order to capture the LH surge also in women with slightly shorter cycles. In the clinic, a positive LH surge is sufficient for diagnosis of an ovulatory cycle, although it is sometimes argued that the final proof of ovulation is the progesterone secretion of the luteal phase (Speroff and Fritz, 2010).

Following ovulation, the dominant follicle develops into a corpus luteum which is capable of estradiol as well as progesterone synthesis (Speroff and Fritz, 2010). Progesterone, at this stage, is needed for endometrial preparation for implantation, in case of conception, and the progesterone peak on menstrual cycle day 21 (or LH +8) coincides with the endometrial implantation window on menstrual cycle day 21 (LH +8) (Nikas and Makrigiannakis, 2003). If LH kits are not used, ovulation can be confirmed by measurement of progesterone. In the clinic, a progesterone serum concentration above 25 nmol/l on cycle day 21 is proof of an ovulatory cycle (Speroff and Fritz, 2010), but progesterone levels >10 nmol/l (taken at some point during the luteal phase) together with a report on normal cycle length can also be used as an indicative of an ovulatory cycle (Nevatte et al., 2013). Although estradiol generally has attracted more scientific attention than progesterone in menstrual cycle studies, it should be noted that the mid-luteal progesterone serum concentration is approximately 100-fold greater than the estradiol levels at the same time-point.

Great inter-individual differences in menstrual cycle length and hormone levels are, however, at hand. In young women, deviations from the typical 28-day menstrual cycle is due to a shorter or prolonged follicular phase (Lenton et al., 1984a), whereas the luteal phase is considered relatively stable with 14 days from the LH surge to onset of menses. In women approaching their forties, shorter menstrual cycle intervals may, however, also be due to a shorter luteal phase as a first sign of ovarian aging, i.e., corpus luteum insufficiency. Short luteal phases occur in approximately 5% of menstrual cycles (Lenton et al., 1984b). Also, great inter-individual variability in hormone levels are typically encountered during the peak hormone phases, and skewed distributions of estradiol and progesterone serum concentrations are typically found, Figure 1.

Estradiol and progesterone are both highly lipophilic and easily pass through the blood-brain barrier. In fact, animal studies and post-mortem studies in reproductive and postmenopausal women indicate that estradiol and progesterone are accumulated in the brain (Bixo et al., 1986, 1995, 1997), with the highest concentration of progesterone found in the amygdala (Bixo et al., 1997). The estradiol receptors (ERα and ERβ) and the progesterone receptors (PRA and PRB) are highly expressed in brain areas associated with reproduction, cognitive function, and emotional processing such as the hypothalamus and the limbic system (for review, see Gruber et al., 2002; Brinton et al., 2008). For example, the expression of the estradiol receptors has been demonstrated in the human amygdala, hippocampus, claustrum, hypothalamus, and the cerebral cortex. Within the human cerebral cortex, the most distinct expression of estradiol receptors is found in the temporal cortex (Osterlund et al., 2000a,b). While human studies are not available for progesterone receptors, animal data suggest that progesterone receptors are also distributed throughout the amygdala, hippocampus, hypothalamus, thalamus, and the frontal cortex (Kato et al., 1994; Guerra-Araiza et al., 2000, 2002, 2003). Additional membrane-bound receptors have emerged as potential mediators of rapid non-genomic effects of estradiol and progesterone in rodent brain, namely G protein-coupled estrogen receptors (GPERs) which are responsive to estradiol in the hippocampus, hypothalamus, cortex and substantia nigra (Hazell et al., 2009). Progesterone, on the other hand, has been reported to bind to the progesterone receptor membrane component 1 (PGRMC1) in the cerebellum, cortical regions, hippocampus, and hypothalamic nuclei (Intlekofer and Petersen, 2011). In addition, progesterone can also be metabolized into neuroactive steroids, among which allopregnanolone and pregnanolone are the two neurosteroids most studied. Neurosteroids potentiate the GABA_A_ receptor, where they increase hyperpolarization and act in a similar manner to barbiturates and benzodiazepines (Melcangi et al., 2011). As GABA is the major inhibitory transmitter in the central nervous system, acute administration of allopregnanolone has sedative, anxiolytic, anti-convulsant properties but may also negatively influence cognitive function (Johansson et al., 2002; Kask et al., 2008a; Melcangi et al., 2011). A functionally relevant amount of allopregnanolone is synthesized in the brain, but the main source of brain and serum allopregnanolone in non-pregnant women is progesterone synthesized by the corpus luteum (Ottander et al., 2005).

## Methods

Over the past years menstrual cycle studies have greatly improved in quality, and strategies and methods for menstrual cycle studies have been established (Becker et al., 2005). Such methods include correct classification of menstrual cycle stage by use of hormonal measures (serum or saliva hormone concentrations, basal body temperature (BBT), or assessments of the LH surge) in addition to calendar-based assessments (Becker et al., 2005). Peripheral concentrations of estradiol and progesterone vary substantially between individuals (Figure 1), why a single measurement alone is insufficient for cycle phase determination. Instead, a combination of calendar method and cycle phase determination is needed. For follicular phase assessments, forward counting from menstrual cycle onset together with a hormonal measurement is sufficient. In the luteal phase, two different approaches are possible: (1) forward counting of days from onset of the LH surge (or BBT rise) or (2) backward counting from onset of next menses together with a progesterone assay. If hormone measures suggest that the cycle phase is incorrect subjects should, of course, be excluded.

In this study we have only accepted studies that have employed some type of hormonal assessment for confirmation of cycle phase according to previous guidelines (Becker et al., 2005). The grand majority of studies included employed either saliva or serum concentrations of hormones, but two studies relied on LH detection only (Epting and Overman, 1998; Pletzer et al., 2011) or basal body temperature (Solis-Ortiz et al., 2004; Solis-Ortiz and Corsi-Cabrera, 2008), respectively. In the manuscripts reviewed, a minority clearly stated that hormonal measurements had been used to exclude subjects who fell out-side of the stipulated cycle phases. For this reason we had to accept all manuscripts that contained some hormonal measurement, although it is unclear if these measures in all cases were acted upon.

The menstrual cycle days reported in each individual study was recalculated according to the idealized 28-day menstrual cycle to facilitate comparison of menstrual cycle stage between studies. Furthermore in this review, early follicular phase is defined as cycle day 1–7, late follicular phase as cycle day 8–14, early luteal phase as cycle day 15–21 and late luteal phase as cycle day 22–28. We have also consistently used the reproductive vocabulary, i.e., early follicular, instead of menstrual, phase.

Results from counterbalanced, longitudinal designs and cross-sectional studies have been separated, with greater emphasis on findings gained by longitudinal studies. Longitudinal studies typically find less pronounced menstrual cycle changes than cross-sectional designs, but are susceptible to training or learning effects (which may be circumvented by counterbalancing the order of study entries). Two of the longitudinal studies included were unbalanced (Becker et al., 1982; Courvoisier et al., 2013), but this has been specifically noted in the results.

The cross-sectional design, on the other hand, is susceptible to selection bias and may end up measuring effects that are more related to inter-individual performance differences than hormonal effects. For this reason the cross-sectional design is associated with an increased risk of chance findings, although this risk can be counteracted by increasing the sample size. With certain experimental set-ups it may, however, be impractical, or sometimes not even possible, to repeat experiments over time.

A meta-analysis was conducted for the error rate in mental rotation tasks by use of the Meta-analysis with Interactive eXplanations (MIX) 2.0 Pro software package. The standardized mean difference in error rate with a 95% confidence interval (CI) was calculated on the basis of mean differences, standard deviations and the number of participants in each of the included studies.

## Cognitive tasks across the menstrual cycle

The predominant hypotheses in the field of menstrual cycle-related cognitive changes state that: (1) Sexually dimorphic cognitive abilities/skills that favor men are improved during phases with low estrogen and progesterone levels such as the early follicular phase, (2) Sexually dimorphic cognitive abilities/skills that favor women are improved during phases with increased estrogen and/or progesterone such as the late follicular phase and mid-luteal phase. Studies along these two hypothesis has been reviewed, although not exclusively so.

### Visuospatial ability

#### Mental rotation

Men outperform women on tasks reflecting visuospatial ability, at least as long as tasks cannot be verbalized (such as object location tasks, small-scale navigation, and landmark-based navigation) (Andreano and Cahill, 2009). The most commonly used test, which also consistently differ between men and women is mental rotation (Andreano and Cahill, 2009). Findings on mental rotation performance across the menstrual cycle are summarized in Table 1. A mentioned concern has been that the mental rotation task should be sufficiently difficult, i.e., three-dimensional instead of two-dimensional depictions and large, as opposed to small, angular disparities should be used in order for menstrual cycle phase differences to be detected (Hausmann et al., 2000; Hampson et al., 2014). As seen in Table 1, because of these concerns, most researchers over the past decade have used the Shepard Metzler or Vandenberg & Kuse mental rotation tasks. While the majority of studies in Table 1 do not demonstrate the expected improved performance in the early follicular phase, or at times of low estradiol levels (Gordon and Lee, 1993; Epting and Overman, 1998; Halari et al., 2005; Schoning et al., 2007; Mordecai et al., 2008; Kozaki and Yasukouchi, 2009; Griksiene and Ruksenas, 2011, however see Hausmann et al., 2000; Maki et al., 2002; Courvoisier et al., 2013; Hampson et al., 2014), significant methodological concerns are at hand in a significant proportion of the studies. For instance, two studies included samples sizes that were extremely small (Hausmann et al., 2000; Dietrich et al., 2001), one study used an unbalanced longitudinal design which may have opened up for training effects (Courvoisier et al., 2013), one study reported on a composite score for visuospatial tasks which may have precluded the detection of more direct effects on mental rotation (Gordon and Lee, 1993), and one study used a task originally developed for children which may have been too easy (Epting and Overman, 1998). In addition, the cross-sectional study by Halari included women in a very narrow time-frame in the follicular phase, whereby the possibility to detect estradiol correlations were in fact minimized (Halari et al., 2005). Finally, two of the studies were neuroimaging studies and it cannot be excluded that behavioral measures in the scanner may differ from that obtained in pure behavioral studies (Dietrich et al., 2001; Schoning et al., 2007). However, even if the studies with methodological concerns are disregarded, four out of the six remaining studies were unable to detect any menstrual cycle differences in mental rotation performance (Maki et al., 2002; Schoning et al., 2007; Mordecai et al., 2008; Kozaki and Yasukouchi, 2009; Griksiene and Ruksenas, 2011; Hampson et al., 2014).

**Table 1:** **Menstrual cycle studies on mental rotation**.

**Authors and design**	**Subjects**	**Cycle phases**	**Task**	**Result**	**Cohen's *d* (error rate)**	**E2 correlations^c^**
**LONGITUDINAL**						
Gordon and Lee, 1993^a^	34 NC/34 OC	2–3/10–14/20–24	Shepard Metzler	No effect of phase or OC		
Epting and Overman, 1998	27 NC	3–4/21–22	Male figures	No effect of phase	0.06	
Hausmann et al., 2000	8 NC	2/22	Vandenberg Kuse	↑ early follicular	0.84	−0.48/−0.70^*^^e^
Dietrich et al., 2001	6 NC	Menses/11–12	Vandenberg Kuse	no effect of Phase		
Maki et al., 2002	16 NC	1–3/19–24	Vandenberg Kuse	↑ early follicular	0.97	−0.51^*^
Schoning et al., 2007	20 NC	1–3/21–25	Vandenberg Kuse	No effect of phase	0.22	
Mordecai et al., 2008	16 NC/20 OC	2–4/20–22	Vandenberg Kuse	No effect of phase or OC	0.03	
Kozaki and Yasukouchi, 2009	16 NC	1–3/high E2	Shepard Metzler	No effect of phase	0.33	
Griksiene and Ruksenas, 2011	20 NC/23 OC	2–5/14/20	Shepard Metzler	No effect of phase or OC^f^		
Courvoisier et al., 2013^b^	7 NC	Once daily 8 weeks	Shepard Metzler	↑ at low E2 phases	0.26	
**CROSS-SECTIONAL**						
Halari et al., 2005^c^	42 NC	3–7	Vandenberg Kuse	No hormonal correlations		−0.29
Hampson et al., 2014^c^^,^^d^	44 NC	Low-E2/highE2	Vandenberg Kuse	↑ low E2	1.14	−0.37^*^
			Clock rotation test, easy	No effect of phase	0.11	
			Clock rotation test, hard	↑ low E2	0.85	−0.38^*^

*NC, normal cycling; OC, oral contraceptive users; E2, estradiol*.

a *A composite score consisting of Shepard Metzler, 3D clocks, point location, and a test of perceptual closure was reported*.

b *Unbalanced design, correlation reported for E2 and error rate*.

c *Partial correlation with control for sex hormone binding globulin (SHBG), otherwise Pearson's correlation*.

d *Low E2 had mean saliva concentration of 3.17 ± 1.0 pg/mL and high E2 6.24 ± 1.7 pg/mL, regardless if tests had been made in the follicular or luteal phases*.

e *Two correlation coefficients were reported, from the first and second test session*.

f *Third generation OC users had longer reaction times in the mental rotation task than normal cycling women, but did not differ in accuracy*.

* *p < 0.05*.

Six of the mental rotation studies provided sufficient information for inclusion in a meta-analytic approach (Epting and Overman, 1998; Hausmann et al., 2000; Maki et al., 2002; Schoning et al., 2007; Mordecai et al., 2008; Kozaki and Yasukouchi, 2009). The meta-analysis yielded an standardized mean difference in error rate of 1.61 (95% CI −0.35 to 3.57, ns), *at present* suggesting no favor of an early follicular phase improvement in mental rotation performance. If the meta-analysis is narrowed down to studies using the Shepard Metzler or Vandenberg & Kuse tasks, this finding remains, standardized mean difference 1.73 (95% CI −0.29 to 3.76, ns). However, it should be noted that while the meta-analysis failed to provide a significant finding, this may very well be due to low power. Another finding that may suggest that further studies in this field should be pursued was that most correlational analyses revealed a negative correlation between mental rotation accuracy and estradiol levels, Table 1.

The neural correlates of mental rotation has been evaluated in relation to the menstrual cycle by two studies (Dietrich et al., 2001; Schoning et al., 2007). Both studies report changes in brain reactivity across the menstrual cycle and an increased reactivity in Brodmann area (BA) 39, or the angular gyrus, during presence of high levels of estradiol. Angular gyrus is involved not only in verbal processing but also in spatial judgment (Chen et al., 2012; Seghier, 2013) and according to the authors, the increased reactivity may reflect an increased need to recruit this area to solve the task at hand during the luteal phase (Schoning et al., 2007; Dietrich et al., 2001).

Yet at the same time, while the hypothesis that mental rotation performance should be superior during phases of low estrogen and progesterone levels could not be substantiated at this stage, future studies may very well alter the picture. There are several reasons for this, first it should be noted that studies that have claimed positive findings almost consistently have reported findings in the same direction, i.e., toward improved mental rotation performance in the early follicular phase. Clearly, adequately powered studies should settle this issue, and by including sufficient information on outcomes, future meta-analyses could, in fact, alter the results of the present review. Secondly, the hypothesis for mental rotation performance may have been too loosely defined. Most studies have used the mid-luteal phase as contrast to the early follicular phase, but at this stage it may be that estradiol levels are not sufficiently elevated for an effect to be noted, or that the mid-luteal progesterone surge counteracts the effect of estradiol. Finally, another interesting aspect is that women with polycystic ovary syndrome, which is characterized by hyperandrogenism (i.e., elevated androgen levels) display superior mental rotation performance in comparison with healthy, naturally cycling women (Barry et al., 2013). Maybe a more male-like performance should be expected not only in the early follicular phase but also among anovulatory women with PCOS, further emphasizing the need to evaluate not only estradiol but also testosterone.

#### Other visuospatial tasks

Findings on a whole range of other tasks evaluating visuospatial ability is presented in Table 2. Besides mental rotation, spatial tests can also broadly be categorized into tasks that evaluate spatial perception and spatial visualization, and among the latter navigation tests and object location test are included (Linn and Petersen, 1985). Notably, while men outperform women on most tasks reflecting visuospatial ability, a female advantage has been noted for tasks that can be verbalized (such as object location) (Andreano and Cahill, 2009). For this reason the hypothesis that visuospatial task performance should be superior in the early follicular phase should not include studies using the object location task, where the opposite hypothesis may be more relevant.

**Table 2:** **Menstrual cycle studies and other visuospatial tests**.

**Authors and design**	**Subjects**	**Cycle phases**	**Task**	**Result**	**Cohen's *d***
**LONGITUDINAL**					
Becker et al., 1982^a^	14 NC	Once daily 2 cycles	Spatial math test	↑ early follicular	
Hampson, 1990	50 NC	3–5/–16	Space relations	↑early follicular^c^	
			Rod and Frame		
			Hidden Figures Test		
Phillips and Sherwin, 1992	25 NC	3–4/19–24	Visual reproduction (VMS)	↑ early follicular	
Gordon and Lee, 1993^b^	34 NC/34 OC	2–3/10–14/20–24	Point Location	No effect of phase or OC	
			Perceptual closure	No effect of phase	
Epting and Overman, 1998	27 NC	3–4/21–22	Rod and Frame	No effect of phase	0.18
			Object location (Silverman)	No effect of phase	0.35
			Water Level	No effect of phase	0.09
Mordecai et al., 2008	16 NC/20 OC	2–4/20–22	Brief Visuospatial Memory Test	No effect of phase or OC	0.13
Solis-Ortiz and Corsi-Cabrera, 2008	9 NC	1–2/13–14/20–21/24–25	Hidden Figures Test	No effect of phase	
			Localization Test	↓ early follicular	
Weis et al., 2011	14 NC	1–3/9–11/21–23	Figure comparison test	No effect of phase	
Hausmann et al., 2000	8 NC	2/22	Hidden Figures Test	No effect of phase	0.13
**CROSS-SECTIONAL**					
Halari et al., 2005	42 NC	3–7	Benton line orientation	No hormonal correlations	
Hampson et al., 2014^d^	44 NC	Low E2/high E2	Perceptual closure	↑ low estradiol	

*NC, normal cycling; OC, oral contraceptive users; E2, estradiol; VMS, Wechsler memory scale*.

a *Unbalanced design*.

b *Composite score consisting of Shepard Metzler, 3D clocks, point location, and a test of perceptual closure was reported*.

c *Composite score of the three tests were used for statistical analyses, a polynomal correlation with E2 was also reported*.

d *Subjects were grouped according to saliva estradiol concentrations, regardless if tests had been made in the follicular or luteal phase*.

However, except for two studies from the same group reporting improved performance on visuospatial performance during the early follicular phase, or at times of low estradiol levels (Hampson, 1990; Hampson et al., 2014), the majority of studies have not been able to discern any menstrual cycle influence on tests of visuospatial memory or ability (Phillips and Sherwin, 1992; Gordon and Lee, 1993; Epting and Overman, 1998; Hausmann et al., 2000; Halari et al., 2005; Mordecai et al., 2008; Solis-Ortiz and Corsi-Cabrera, 2008; Weis et al., 2011), Table 2. Again, a number of methodological concerns have been identified in the studies; several studies suffer from low power (Hausmann et al., 2000; Solis-Ortiz and Corsi-Cabrera, 2008), two studies report on a visuospatial composite score (Hampson, 1990; Gordon and Lee, 1993), and one study involved repeated, daily testings during two menstrual cycles opening up for practice effects (Becker et al., 1982). Furthermore, because of the variety of tasks reflecting various measure of spatial perception and spatial visualization, no attempt for meta-analysis was made. Among the studies that reported a menstrual cycle influence, Hampson (1990) found improved visuospatial ability in the menstrual phase using a composite score of three different tasks (Hampson, 1990). Similarly, Hampson et al. (2014) evaluated visuospatial abilities, including mental rotation, in women with low (approximately corresponding to early follicular phase levels) and high (approximately corresponding to late follicular levels) estradiol levels, regardless if subjects had been assessed in the follicular or luteal phases of the menstrual cycle (Hampson et al., 2014). While this may be a biologically sound approach, it also serves as an example that perhaps no predictive menstrual cycle-related effects in visuospatial abilities exist, as low estradiol levels can be found during the early follicular phase, post-ovulation, the late luteal phase and during anovulatory cycles.

It may also be argued that certain math tests involve components of visuospatial ability. Two studies have evaluated such tests across the menstrual cycle (Becker et al., 1982; Pletzer et al., 2011). Although both studies reported on superior performance in the follicular phase compared to the luteal phase, the study by Becker and colleagues had an unbalanced longitudinal design, and Pletzer and colleagues did not distinguish between the early and late follicular phase, hence the role of low estradiol was not entirely captured (Becker et al., 1982; Pletzer et al., 2011). Performance on simple mathematical calculations such a subtraction and multiplication appear not to differ across cycle phases (Hampson, 1990). No studies on menstrual cycle influence on virtual and real world navigation have thus far been reported.

### Verbal tasks

A female advantage for verbal fluency and verbal memory is well documented (Andreano and Cahill, 2009). Accordingly, the predominant hypothesis for menstrual cycle studies on verbal fluency and memory has thus been that women should perform better at these tasks during time-periods of high estradiol levels, i.e., during the late follicular phase or mid-luteal phase.

The review of menstrual cycle studies on verbal tasks is summarized in Table 3. Again, a number of studies have methodological flaws including low power (Rosenberg and Park, 2002; Konrad et al., 2008; Solis-Ortiz and Corsi-Cabrera, 2008), multiple test sessions in individual subjects (Rosenberg and Park, 2002; Solis-Ortiz and Corsi-Cabrera, 2008), composite scores by which specific task performance may be disguised (Gordon and Lee, 1993), or used unwisely chosen time-frames for their assessments (Halari et al., 2005). The most frequently used tasks include verbal fluency and verbal recall, but tests reflecting semantic retrieval and implicit verbal memory have also been employed. As seen in Table 3, relatively few studies have documented any significant findings across the menstrual cycle and no consistent pattern, according to the above hypotheses, emerges (Hampson, 1990; Phillips and Sherwin, 1992; Gordon and Lee, 1993; Maki et al., 2002; Rosenberg and Park, 2002; Halari et al., 2005; Konrad et al., 2008; Mordecai et al., 2008; Solis-Ortiz and Corsi-Cabrera, 2008; Hatta and Nagaya, 2009; Griksiene and Ruksenas, 2011; Jacobs and D'Esposito, 2011; Hampson et al., 2014). Two imaging studies have evaluated verbal tasks across the menstrual cycle. In line with the behavioral results, Rumberg and colleagues found no difference in brain activation during verb generation between women examined in the early follicular and late follicular/early luteal phase (Rumberg et al., 2010). Dietrich, using a similar task, on the other hand reported on increased activation of a number of language related areas (BA 45/46, 6, and 40) during the late follicular phase in comparison with the early follicular phase (Dietrich et al., 2001).

**Table 3:** **Menstrual cycle studies on verbal skills and verbal memory**.

**Authors and design**	**Subjects**	**Cycle phases**	**Task**	**Result**	**Cohen's *d***
**LONGITUDINAL**					
Gordon and Lee, 1993^a^	34 NC/34 OC	2–3/10–14/20–24	Verbosequential score	No effect of phase or OC	0.05
Griksiene and Ruksenas, 2011	20 NC/23 OC	2–5/14/20	Verbal fluency	No effect of phase, NC > OC	
Hampson, 1990	50 NC	3–5/–16	Verbal fluency	No effect of phase	
Hatta and Nagaya, 2009	30 NC	2–3/21–22	Verbal recall	No effect of phase	0.23
Jacobs and D'Esposito, 2011^b^	24 NC	1–2/11–12	Verbal working memory	No effect of phase	
Konrad et al., 2008	12 NC	1–3/19–23	Synonym generation	No effect of phase	0.04–0.23
Maki et al., 2002	16 NC	1–3/19–24	Verbal fluency	↑ midluteal	0.56
			Implicit verbal memory	↑ midluteal	0.72
			Explicit verbal memory	No effect of phase	−0.45
Mordecai et al., 2008	16 NC/20 OC	2–4/20–22	CVLT verbal recall	No effect of phase	0.30
				↑ active treatment in OC	
			Verbal fluency	No effect of phase or OC	0.04
Phillips and Sherwin, 1992	25 NC	3–4/19–24	Immediate paragraph recall	No effect of phase	
			Delayed paragraph recall	No effect of Phase	
			Associate verbal recall	No effect of Phase	
			Digit span	No effect of phase	
Solis-Ortiz and Corsi-Cabrera, 2008	9 NC	1–2/13–14/20–21/24–25	Verbal fluency	↑ late follicular vs. late luteal	
Rosenberg and Park, 2002	8 NC/10 OC	0, 7, 14, 21	Verbal working memory	↑ day 7 and 14 vs. day 0 and 21	
				No effect of OC	
**CROSS-SECTIONAL**					
Hampson et al., 2014^c^	44 NC	lowE2/highE2	Rhyme generation	No effect of phase	0.21
			Synonym generation	No effect of phase	−0.18
Halari et al., 2005	42 NC	3–7	Verbal fluency	No hormonal correlation	

*NC, normal cycling; OC, oral contraceptive users; E2, estradiol; CVLT, California Verbal Learning Test*.

a *Composite score consisting of perception, verbal recall and verbal fluency*.

b *A COMT genotype by phase interaction was reported*.

c *Subjects were grouped according to saliva estradiol concentrations, regardless if tests had been made in the follicular or luteal phase*.

However, two important points should be stressed regarding verbal skills across the menstrual cycle. First, two studies have evaluated verbal working memory, which also taps into prefrontal dopaminergic function. The Rosenberg study, albeit small in sample size, is one of few studies which demonstrated improved performance during phases of the menstrual cycle that are characterized by high estrogen levels (Rosenberg and Park, 2002). In addition, while the overall cycle effect was negative, Jacobs and D'Esposito, in fact, demonstrated that verbal working memory task performance was modulated by an interaction between *COMT* Val158Met and estradiol levels (Jacobs and D'Esposito, 2011). In the presence of high late follicular phase estradiol levels, an improved cognitive performance was found in Val/Val carriers (which putatively is associated with lower frontal dopamine levels), whereas a deteriorated performance was found in Met/Met carriers in comparison with the performance during the early follicular phase (Jacobs and D'Esposito, 2011). Thus, verbal working memory seems to be one verbal task were estradiol load is important, however, the genetic make-up of women may also influence the outcome. Further discussion of tasks that probe the prefrontal lobe are found below.

Secondly, although the menstrual cycle studies on verbal memory were mostly negative it should be noted that this does not rule out the possibility of an estradiol influence. Verbal memory is a relatively common cognitive outcome in randomized clinical trials on estrogen treatment in postmenopausal women, and possibly the only cognitive task where there is some evidence that estrogen treatment may have a beneficial effect. According to a recent review, there is some evidence that estrogen treatment protects verbal memory in surgically postmenopausal women (Sherwin, 2012), whereas it has no effect when initiated more than a decade after the menopause. Possibly the hormonal changes across the menstrual cycle are too swift to detect an impaired performance in the relatively short early follicular phase, especially since extremely low estradiol levels (in the postmenopausal range) not are seen in all women, and if seen, only for a few days. Evidence for this assumption may be drawn from a study on young women treated with gonadotropin releasing hormone agonists, which resulted in suppressed estradiol levels. Following eight weeks of treatment, the estradiol suppression obtained was associated with impaired verbal memory performance (Craig et al., 2007).

### Cognitive control

Given the suggested modulatory role for estrogen (and progesterone) in the frontal dopaminergic system (reviewed in Becker and Hu, 2008), studies probing cognitive control across the menstrual cycle have also been reviewed. Cognitive control has been evaluated by use of the Wisconsin Card Sorting Test and various inhibitory tasks. While Solis-Ortiz and co-workers initially reported on improved performance in the Wisconsin Card Sorting Test during the early follicular and early luteal phase (Solis-Ortiz et al., 2004), they later failed to replicate this finding (Solis-Ortiz and Corsi-Cabrera, 2008). Using tasks that test the ability to inhibit prepotent responses, Colzato et al. reported on less efficient inhibition in the late follicular phase (Colzato et al., 2010), whereas Bannbers and colleagues found no effect of menstrual cycle, either in accuracy or reaction time to a Go-NoGo task (Bannbers et al., 2012). However, using a task that probed inhibitory *input* control, as opposed to the Stop-signal task and Go-NoGo concerned with inhibitory *output* control, Colzato and colleagues demonstrated superior inhibition of return in the late follicular phase (Colzato et al., 2012). Also, the tendency to choose an immediate reward over greater, delayed rewards reportedly decrease between the early and late follicular phase, and this decrease is influenced by estradiol levels (Smith et al., 2014). Finally, working memory is also a prefrontal cortex-dependent cognitive function that supports an array of essential human behaviors. As already mentioned in the section on verbal tasks, verbal working memory performance appear superior at times of high estradiol levels (Rosenberg and Park, 2002; Jacobs and D'Esposito, 2011), and this may also be true for spatial working memory (Hampson and Morley, 2013).

## Emotional aspects of the menstrual cycle

Over the past years, the menstrual cycle influence on emotional processing, emotional memory, and fear conditioning has gained increasing interest. While findings thus far are scarce, this line of research taps into the emotional disorders of the menstrual cycle.

Many women suffer from emotional problems during the menstrual cycle. Approximately 2–10% of women in child-bearing ages are afflicted by severe premenstrual symptoms, and 2–5% fulfill criteria for premenstrual dysphoric disorder (PMDD) (O'Brien et al., 2011). Population-based epidemiological studies furthermore suggest that sub-threshold PMDD may be even more prevalent, found in 18% of women (Wittchen et al., 2002). PMDD is typified by socially disrupting symptoms such as depressed mood, anxiety, and irritability which consistently appear in the late luteal phase of the menstrual cycle and remit 1–2 days after onset of menses (O'Brien et al., 2011). Premenstrual emotional disturbances have consistently been linked to progesterone exposure during the luteal phase, as symptom remit during GnRH agonist suppression of ovarian hormone levels, and are reinstated with progesterone add-back (reviewed in Nevatte et al., 2013). However, the hormone fluctuations of the menstrual cycle may also be of importance for major depressive disorder and a number of anxiety disorders, as the female bias for developing these disorders appear at menarche (suggesting activational effects of ovarian steroids) (Kessler et al., 1993), but also because many women with these disorders complain of a premenstrual worsening (Sigmon et al., 2000; Kornstein et al., 2005; Nillni et al., 2012). Overall, besides the mate preference studies (which is beyond the scope of this review, see instead Gangestad and Thornhill, 2008; Jones et al., 2008; Little, 2013), the emotional processing aspects of the menstrual cycle are far less researched than the cognitive aspects, Table 4. Notably, most efforts in this area has had a cross-sectional approach, and only two longitudinal studies have been identified (Conway et al., 2007; Bayer et al., 2014).

**Table 4:** **Menstrual cycle studies on emotion processing**.

**Authors and design**	**Subjects**	**Cycle phases**	**Design**	**Task**	**Result**
**EMOTION RECOGNITION**					
Derntl et al., 2013	37 NC	2–5/18–25	c.s	Facial emotion recognition	↓ Accuracy mid-luteal phase
				Affective responsiveness	↓ reaction times mid-luteal phase
Conway et al., 2007	52 NC	Low P/high P	Long	Facial emotion recognition (averted gaze)	↑ fearful faces perceived as more intense in high P
Derntl et al., 2008a	32 NC	7–13/15–27	c.s	Facial emotion recognition	↓ Overall accuracy luteal phase
				Memory of emotion recognition	No difference across cycle phases
Guapo et al., 2009	30 NC	1–5/12–14/21–23	c.s	Facial emotion recognition	↓ Accuracy mid-luteal phase for sad faces
van Wingen et al., 2007	16 NC	Day 2–7	P treatment	Facial recognition	↓ Accuracy in P-treated
Gasbarri et al., 2008	56 NC	1–2/4–13/14–32	c.s	Working memory for facial emotion recognition	↓ Accuracy for sad and disgusted faces in late follicular phase
**EMOTIONAL MEMORY**					
Bayer et al., 2014	22 NC	1–4/17–23	Long	Emotional memory (IAPS)	↓ Midluteal recognition for negative items
Ertman et al., 2011	60 NC	1–14/15–28	c.s.	Emotional memory (IAPS)	↑ Luteal free recall of negative items
Nielsen et al., 2013	42 NC/36 OC	1–14/15–28	c.s.	Emotional memory (narrative)	↑ Luteal memory for peripheral details
Andreano et al., 2008	64 NC	1–7/8–13/18–24	c.s.	Emotional memory (narrative) ± CPS	No effect of stress or cycle phase on recall
Felmingham et al., 2012	56 NC	High P/low P	c.s.	Emotional memory (IAPS) ± CPS	↑ memory during stress in high P
Kuhlmann and Wolf, 2005	27 NC/20 OC	2–4/20–24	Cortisol/placebo	Emotional memory (negative/neutral words)	No difference in cortiol-induced retrieval impairment
**FEAR LEARNING**					
Merz et al., 2012	60 NC/30 OC	3–8/20–26	c.s	Fear conditioning + cortisol/placebo	No difference across cycle phases
Milad et al., 2010	36 NC	High E2/low E2	c.s	Fear conditioning + fear extinction	No difference between groups
Zeidan et al., 2011	34 NC	High E2/low E2	c.s	Fear conditioning + fear extinction	No difference between groups
				Fear extinction recall	↓ recovery of fear in high E2
Graham and Milad, 2013	31 NC	Day 1–5	E2 treatment	Fear conditioning + fear extinction	No difference
				Fear extinction recall	↓ recovery of fear in E2-treated
**SPONTANEOUS INTRUSIVE RECOLLECTIONS**					
Ferree et al., 2011	54 NC	1–13/15–28	c.s	Film clips	↑ SIR in early luteal phase
Soni et al., 2013	41 NC	7–11/16–20/24–28	c.s	Film clips	↑ SIR in early luteal phase

*NC, normal cycling; OC, oral contraceptive users; E2, estradiol; P, progesterone, c.s., cross-sectional; long, longitudinal-balanced; SIR, Spontaneous intrusive recollections;, IAPS, International Affective Picture System; CPS, cold pressor stress*.

A number of studies have investigated how facial emotion recognition is affected by the menstrual cycle, Table 4. Compared to the midluteal phase, a better emotion recognition accuracy has been suggested in the early follicular phase (Derntl et al., 2013) and late follicular phase (Derntl et al., 2008a,b) independent of emotion stimuli, or specifically for sad faces (Guapo et al., 2009). However, when working memory for facial emotion recognition was tested, worse performance for sad and disgusted faces was detected in the early follicular phase (Gasbarri et al., 2008), possibly because the test incorporated cognitive aspects also influenced by estrogen. Hence, emotion recognition accuracy appear poorer in the luteal phase, specifically for the negative emotional stimuli (Derntl et al., 2008b) and these findings are corroborated by a report on decreased facial recognition accuracy upon acute progesterone administration (van Wingen et al., 2007). Women also demonstrate a greater tendency to perceive fearful expressions (with averted as compared to direct gaze) as more intense if progesterone levels are high (Conway et al., 2007), and respond faster to sad and angry situations or sad faces in the mid-luteal phase (Gasbarri et al., 2008; Derntl et al., 2013), as well as to other aversive stimuli, such as snakes (Masataka and Shibasaki, 2012). However, all of these studies have been performed in healthy women and it is unclear as to what extent premenstrual disorders influence the overall results of these studies. While most studies used psychiatric interviews to exclude ongoing depressive and anxiety disorders, only one study utilized daily symptom scoring for diagnosis of PMDD, and consequently, exclusion of PMDD in the control group (Rubinow et al., 2007). Not surprisingly, women with PMDD showed impaired facial emotion recognition performance and a negative bias (neutral faces being misjudged as sad) in the luteal phase, whereas the controls did not differ across cycle phases (Rubinow et al., 2007). Besides this finding, there is plenty of evidence to suggest that PMDD is associated with altered emotional processing across the menstrual cycle (Epperson et al., 2007; Kask et al., 2008b; Bannbers et al., 2011; Gingnell et al., 2012, 2013a, 2014; Hoyer et al., 2013; Comasco et al., 2014). Clearly, these findings demonstrate that premenstrual disorders need to be accounted for in menstrual cycle research on emotion processing.

An increasing interest in menstrual cycle influence on emotional memory has also been noted, Table 4. Emotional memory depends on hypothalamus-pituitary-adrenal (HPA) axis hormones and sympathetic activity, and a sex-related difference has been suggested (Andreano and Cahill, 2009). Again, progesterone, and the luteal phase has attracted most attention, presumably as progesterone in other species is considered a stress hormone (Fajer et al., 1971; Frye, 2007; Axner, 2008), and because of the close relationship between progesterone and the HPA axis, notably most commonly studied in relation to onset of human labor (Vrachnis et al., 2012). Across the menstrual cycle, baseline cortisol levels appear unaltered (Nepomnaschy et al., 2011), whereas cortisol reactivity to stress seem elevated in the luteal phase (Kirschbaum et al., 1999).

Results on emotional memory throughout the menstrual cycle relatively consistent. Whereas the only longitudinal study in the field recently reported decreased recognition for negative items in the luteal phase (Bayer et al., 2014), cross-sectional studies have demonstrated no difference (Felmingham et al., 2012) or enhanced memory for emotional items (Ertman et al., 2011) in the luteal phase. Furthermore, enhanced memory for peripheral details of an emotional story has been linked to the luteal phase (Nielsen et al., 2013) and emotional memory correlated positively with progesterone levels sampled at the time of encoding (Ertman et al., 2011).

Spontaneous intrusive recollections (SIR) are known to follow emotional events in clinical and non-clinical populations, among the former, posttraumatic stress disorder is the most obvious example. Indeed, also the menstrual cycle may influence such recollections in trauma patients (Bryant et al., 2011) and as well as in healthy women (Ferree et al., 2011; Soni et al., 2013). These flashbacks or spontaneous intrusive recollections appear to be more common if the trauma or experimental exposure to aversive stimuli occurred in the luteal phase (Bryant et al., 2011; Ferree et al., 2011; Soni et al., 2013), and again, progesterone levels were positively correlated with SIR frequency (Ferree et al., 2011).

In addition, various strategies to modulate the HPA axis across the menstrual cycle have been employed, for instance by cortisol treatment or by use of stress tests such as the Cold Pressor Stress test (CPS). Kuhlmann and colleagues reported that cortisol treatment resulted in impairment of emotional verbal memory, but found no difference in the cortisol-induced memory impairment between women assessed in the follicular or luteal phases (Kuhlmann and Wolf, 2005). While one study reported that women with high progesterone levels had greater memory recall for negative images if subjected to post-training CPS physical stress (Felmingham et al., 2012), others found no difference across cycle phases in memory performance following CPS (Andreano et al., 2008). Andreano and colleagues, however, demonstrated that post-CPS cortisol was correlated with memory retrieval in the luteal phase although not in the follicular phase (Andreano et al., 2008).

Fear conditioning is often used as a model for the formation of emotional memories (LeDoux, 2000). By using a 2-day fear conditioning paradigm consisting of fear conditioning, and extinction learning on the first day, and extinction recall and fear renewal on the second day, Milad and others have over time presented relatively consistent results as to the estradiol involvement in consolidation or maintenance of extinction, whereas fear acquisition and fear extinction appear not to be influenced by hormonal state (Milad et al., 2010; Zeidan et al., 2011; Merz et al., 2012; Graham and Milad, 2013), Table 4. Their studies have suggested greater extinction memory (i.e., less recovery of fear upon fear renewal) in women during the late follicular phase or in women with high estradiol levels (irrespectively of cycle phase) (Milad et al., 2010; Zeidan et al., 2011; Graham and Milad, 2013), corroborated by the finding that a single estradiol administration during the early follicular phase resulted in similarly enhanced consolidation of extinction (Graham and Milad, 2013). No effect of progesterone or the luteal phase was noted (Milad et al., 2010; Zeidan et al., 2011). Thus, maintenance of fear extinction which is an important goal of cognitive behavioral therapy appears superior if applied in the follicular rather than in the luteal phase.

Clearly, all of these findings point toward altered emotion processing across the menstrual cycle, which is in line with the clinically relevant emotional disturbances that are reported by a substantial fraction of women. Further studies in this area could help explain the underlying mechanisms of emotional problems in the luteal phase. However, longitudinal studies are awaited, and preferably authors should investigate to which extent also PMDD (or sub-threshold PMDD) influence their results. This could be relatively easily achieved, for instance by asking women to keep daily prospective records of mood symptoms throughout the menstrual cycle in which they are tested.

The luteal phase is dominated by elevated progesterone and estradiol, and without hormonal interventions, it may be difficult to disentangle which of these two hormones is driving the results. For instance, while it is generally accepted that progesterone is the symptom provoking hormone in PMDD, hormone interventions have suggested that also estradiol plays a role. Segebladh and colleagues treated PMDD women with GnRH agonists for hormone suppression and evaluated the return of symptoms when women were exposed to three different hormonal treatments. The combination of a high estrogen dose (and progesterone) was more symptom provoking than a low estrogen dose together with progesterone (Segebladh et al., 2009). Possibly, as one of the most important aspects of estrogen action (in the brain and elsewhere) is to up-regulate progesterone receptors, increased availability to estrogen may thus result in more progesterone receptors for progesterone to act upon.

Progesterone is also associated with more complex actions than estradiol. On the one hand it has been associated with anxiolytic and sedative effects, on the other hand also with anxiogenic and depressive states. For the interested reader, several reviews on these conflicting aspects of progesterone action are available (Sundstrom Poromaa et al., 2003; Backstrom et al., 2011). In addition, it should be pointed out that women with premenstrual disorders experience their most intense symptoms in the late luteal phase, when progesterone levels are declining, not at the progesterone peak (Nevatte et al., 2013).

### fMRI studies on emotion processing during the menstrual cycle

Neuroimaging techniques, such as functional magnetic resonance imaging (fMRI), are useful tools to gather further insight into CNS processing. The effects of the menstrual cycle and other hormone interventions during both emotional and cognitive tasks was recently reported (Toffoletto et al., 2014), and this review merely highlights studies which correspond to previously reported behavioral data, Table 5. Most studies have used a longitudinal approach with repeated scanning sessions in the same participants, but the use of different tasks and contrasts for generation of the fMRI results, as well as variations in time points for assessments hampers the comparability of studies. However, some tentative conclusions may be drawn.

**Table 5:** **Menstrual cycle studies including fMRI with emotional stimuli**.

**Author**	**Subjects**	**Cycle phases**	**Task**	**Behavioral results**	**Contrast**	**ROI analyses**	**Whole Brain analyses**
Gingnell et al., 2013a	14 NC	6–12/22–27	Neg. and pos. images and the anticipation thereof	No effect of phase	Neg. > pos.	n.a.	None
Protopopescu et al., 2005	12 NC	8–12/–23–27	Go NoGo emotional words	n.a.	Neg. Go > neu Go	n.a.	↑↓ OFC in the luteal phase
					Pos. Go > neu Go	n.a.	↑ ACC in the luteal phase
					Neg. NoGo > neu NoGo	n.a.	↑ OFC in the luteal phase
Gingnell et al., 2012	15 NC	6–12/22–27	Facial emotion recognition	No effect of phase	Angry & afraid faces > shapes	↑ amygdala in luteal phase	n.a.
Gingnell et al., 2013b	17 NC	1–10/15–21	Facial emotion recognition	no effect of phase	Angry & afraid faces > shapes	↓ amygdala in luteal phase	n.a.
Andreano and Cahill, 2010	17 NC	1–7/18–24	Neg. and neutral images	n.a.	Neg. > neutral	↑ amygdala and hippocampus in luteal phase	↑ IFG, fusiform gyrus, cerebellum, CN in the luteal phase
Goldstein et al., 2005	12 NC	2–3/16–18	Neg. and neutral images	n.a.	Neg. > neutral	↑ BA 40, BA 423 in late follicular	n.a.
						↓ ACC, hypothalamus, brain stem, OFC, amygdala, BA 47, BA 10, BA 18, BA 19, BA 37, BA 30, BA 23, BA 39, cerebellum in late follicular	
Bayer et al., 2014	22 NC	0–4/17–23	Encoding of neg., pos. and neutral images	↓ recollection of negative stimuli in luteal phase	Emotional (hit>miss) > neutral (hit>miss)	↓ hippocampus in luteal phase	n.a.
					Negative (hit>miss) > neutral (hit>miss)	↑ amygdala in luteal phase	n.a.
					Positive (hit>miss) > neutral (hit>miss)	↓ ACC in the luteal phase	n.a.
Rupp et al., 2009	10 NC	10–12/19–23	Evaluation of attractiveness in houses and faces	No effect of phase	Faces > houses	n.a.	↓ OFC in luteal phase
**CROSS-SECTIONAL**							
Derntl et al., 2008a	22 NC	1–14/15–28	Facial emotion recognition	↓ recognition accuracy in luteal phase	Emotional faces > cross hair	↓ amygdala in the luteal phase	↓ hippocampus in the luteal phase
					Disgusted faces > cross hair	n.a.	↓ fusiform gyrus in the luteal phase
					Sad faces > cross hair	n.a.	↓ MTG in the luteal phase
					Neutral faces > cross hair		↓ hippocampus in the luteal phase
Zeidan et al., 2011	34 NC	Low E2/High E2	Fear conditioning and extinction	No effect of group	CS+ > CS-	None	n.a.
				No effect of group	Late CS+E > early CS+E	↑ mPFC in high E2	n.a.
				↑ extinction memory in high E2	first CS+E >first CS+NE	↑ mPFC and amygdala in high E2	n.a.

*NC, normal cycling; E2, estradiol; neg., negative; pos., positive; neu, neutral; ACC, anterior cingulate cortex; dmPFC, dorsomedial prefrontal cortex; dlPFC, dorsolateral prefrontal*.

A pattern of increased amygdala reactivity to negative emotional stimuli in the luteal phase appears in several (Andreano and Cahill, 2010; Gingnell et al., 2012; Bayer et al., 2014), but not in all (Gingnell et al., 2013a,b) studies. A sensitivity in the amygdala to increases in progesterone is also supported by the increased reactivity in the amygdala, fusiform gyrus, inferior frontal gyri, cerebellar vermis, and supplementary motor area observed after acute progesterone administration (van Wingen et al., 2008). It should also be noted that the decrease in amygdala reactivity reported by Gingnell et al. is most likely due to habituation, as this study was not counterbalanced for phase of entry (Gingnell et al., 2013b). One cross-sectional study by Derntl et al. (2008a) report the opposite pattern with amygdala reactivity to facial stimuli being higher in the follicular than in the luteal phase. Being one of the core structures in the fear network (Shin and Liberzon, 2010) the sensitivity in the amygdala to changes in ovarian steroid hormones across the menstrual cycle fits nicely with the increased susceptibility to anxiety and depressive symptoms in the luteal phase. Two studies have assessed brain reactivity during positive stimuli and do not report menstrual cycle differences in amygdala reactivity, but increases in the ACC reactivity during the luteal phase (Protopopescu et al., 2005; Amin et al., 2006). In response to negative emotional stimuli, the ACC reactivity has been reported to decrease in the late follicular phase (Goldstein et al., 2005).

Bayer et al. reported impaired recognition of negatively valenced stimuli in the mid luteal phase, but otherwise no differences in behavior have been reported in the longitudinal fMRI studies evaluating menstrual cycle effects (Bayer et al., 2014). One reason for this may of course be lack of power. Due to costly and time-consuming procedures, imaging studies tend to include few participants. It may also be that brain reactivity represents a more sensitive measure of the ovarian steroid hormone influence. This is in parallel to the increased emotion-induced amygdala reactivity found in non-depressed, non-anxious healthy carriers of the short version of the serotonin transporter promoter length polymorphism (Hariri et al., 2002; Fakra et al., 2009). However, the lack of differences in behavioral measures may also be due to the fact that healthy women are capable of using compensatory mechanisms to adjust for the different hormonal exposures. This further highlights the importance of the chosen paradigms for fMRI and that due care should be made to assure that the used stimuli is relevant to the mechanism that is to be studied.

In conclusion, the hitherto performed fMRI-studies rarely report of behavioral differences across phases, but indicate that brain reactivity may differ. The most consistent finding so far appears to be an increased amygdala response to negative emotional stimuli in the luteal phase, and an increase in ACC reactivity during the processing of positive stimuli in the luteal phase, but the lack of replications, and differences in used paradigms limits the conclusions that can be drawn.

## Conclusion

The menstrual cycle remains an intriguing, natural experiment of relevance to many researchers in medical and psychological disciplines. While the earliest reports on menstrual cycle findings were devoted to explore the suitability of women in work-life and areas dominated by males (Sommer, 1973), the past years research appear driven by the increasing interest in sex influences on neurobiology. However, despite its' immediate appeal and accessibility, menstrual cycle studies require skillful and meticulous handling (Becker et al., 2005), and positive findings have in many cases turned out to be notoriously difficult to replicate.

According to this review, the best evidence suggest that differences across the menstrual cycle in sexually dimorphic tasks, such as mental rotation, visuospatial ability, verbal memory and verbal fluency, are small and difficult to replicate. This finding is partly in line with previous reviews which have either suggested no influence of the menstrual cycle (Sommer, 1973), or some influence although at the same time emphasizing that such changes would not be clinically relevant (Sherwin, 2012). Also, in perspective of the difficulties in establishing a firm role for estrogen treatment on cognitive function in postmenopausal women (Sherwin, 2012) (some evidence suggest a positive influence by estrogen on verbal memory if estrogen treatment is initiated in close temporal relationship with the menopause, Sherwin, 2012), it may not come as a surprise that menstrual cycle studies have, for the most part, failed to prove a menstrual cycle influence on cognitive function. Factors contributing to this may include the younger age of women included in menstrual cycle studies (assuming that a positive or protective effect of estrogen would be more difficult to detect in young women due to roof effects, i.e., a majority of women investigated at an age when cognitive decline has not yet set in), or that longer exposure of estrogen (or estrogen deficiency) is needed for detection of any cognitive effects, or that the grand majority of menstrual cycle studies have been underpowered to detect the presumably relatively small or modest effect sizes across cycle phases. Another reason why findings in menstrual cycle studies probing cognitive function have been difficult to replicate may also be the genetic make-up of women. Recently, Jacobs and D'Esposito demonstrated that prefrontal cortex activation in relation to a working memory task was modulated by an interaction between *COMT* Val158Met and estradiol levels (Jacobs and D'Esposito, 2011). Similarly, the tendency to choose an immediate reward over greater, delayed rewards decreases between the early and late follicular phase, and this decrease was influenced by estradiol levels and driven by Val/Val carriers of the *COMT* Val158 Met genotype (Smith et al., 2014). Finally, in line with the above findings, yet another reason for inconsistent menstrual cycle effects could be that estradiol and/or progesterone act as modulators for other, classical neurotransmitters. For instance, in the above cited studies, the effect of estradiol putatively depends on frontal dopamine levels. Given the wide-spread interactions of estradiol and progesterone on the serotonin neurotransmitter system (Bethea et al., 2002), GABA (Sundstrom Poromaa et al., 2003), and neuropeptides such as brain-derived neurotrophic factor (Comasco et al., 2014), additional complexity is brought into the picture. Disentangling these relationships may help in in understanding why behavioral effects of ovarian steroids are detected only inconsistently.

Further studies on the menstrual cycle modulation of emotional processing are also of importance, as they may ultimately be relevant to women's mental health. Premenstrual disorders, i.e., premenstrual syndrome and premenstrual dysphoric disorder are relatively common in women of fertile ages, and represent a great burden for afflicted women, often associated with impaired social or work-related functioning. This review has emphasized that the luteal phase is associated with impaired emotion recognition accuracy (Conway et al., 2007; van Wingen et al., 2007; Derntl et al., 2008b, 2013; Gasbarri et al., 2008; Guapo et al., 2009) and enhanced emotional memory. Emotional events that occur during the luteal phase more often result in spontaneous intrusive recollections (Ferree et al., 2011; Soni et al., 2013), and traumatic flashback memories are more common when the trauma takes place in the luteal phase (Bryant et al., 2011). In addition, a number of studies have pin-pointed progesterone as the driving factor for these findings; progesterone levels correlated with emotional memory (Ertman et al., 2011) and positively predicted intrusive memories (Ferree et al., 2011). A number of imaging studies have also reported on increased luteal phase reactivity in core structures of the fear network such as amygdala (Andreano and Cahill, 2010; Gingnell et al., 2012; Bayer et al., 2014) and the anterior cingulate cortex (Protopopescu et al., 2005; Amin et al., 2006). Again, progesterone appear a key factor for this finding as increased amygdala reactivity became evident following progesterone administration in the early follicular phase (van Wingen et al., 2008). Taken together, these findings suggest that progesterone, or at least the combined effect of estradiol and progesterone of the luteal phase, have the ability to influence various aspects of emotional processing, which may have repercussions for the clinical presentation of emotional disturbances in the luteal phase. Further studies in this area are awaited.

In conclusion, to the extent that behavioral changes have been demonstrated over the course of the menstrual cycle, the best evidence suggests that differences in sexually dimorphic tasks are small and difficult to replicate. However, emotion-related changes are more consistently found, and are better associated with progesterone than with estradiol, such that high progesterone levels are associated with increased amygdala reactivity and increased emotional memory.

### Conflict of interest statement

Inger Sundström-Poromaa serve occasionally on advisory boards or act as invited speaker at scientific meetings for MSD, Bayer Health Care, Novo Nordisk, and Lundbeck A/S. The authors declare that the research was conducted in the absence of any commercial or financial relationships that could be construed as a potential conflict of interest.
